# Evaluating fluoride-related YouTube videos in Japan: A comparative analysis of understandability, actionability, and reliability between pro- and anti-fluoride content

**DOI:** 10.1016/j.pecinn.2026.100458

**Published:** 2026-02-08

**Authors:** Hikari Sophia Nagao, Tsuyoshi Okuhara, Emi Furukawa, Hiroko Okada, Takahiro Kiuchi

**Affiliations:** a Department of Health Communication, Graduate School of Medicine, The University of Tokyo, Japan; b Department of Health Communication, School of Public Health, Graduate School of Medicine, The University of Tokyo, Japan; c University Hospital Medical Information Network (UMIN) Center, The University of Tokyo Hospital, Japan

**Keywords:** YouTube, Fluoride, Health information, Misinformation, Health communication

## Abstract

**Objective:**

This study aimed to compare pro- and anti-fluoride Japanese YouTube videos on understandability, actionability, flow, reliability, and engagement.

**Methods:**

Eighty-four videos found via a keyword search (pro = 49, anti = 18, other = 17) were analyzed. Quality was assessed using three validated tools—namely, Patient Education Materials Assessment Tool for Audiovisual Materials (PEMAT-A/V), Global Quality Score (GQS), and modified DISCERN (mDISCERN). Engagement was measured as view rate.

**Results:**

Pro-fluoride videos scored higher on reliability (mDISCERN 2.6 ± 1.0 vs 1.3 ± 0.6) and overall quality (GQS 2.5 ± 1.4 vs 1.0 ± 0.9; both *p* < 0.001). No group differences emerged for understandability (58% versus 53%; *p* = 0.23) or actionability (60% versus 53%; *p* = 0.33). In the anti-fluoride group, a higher view rate positively correlated with understandability and GQS (ρ ≈ 0.53; p ≈ 0.03); no correlation was found for pro-videos. Only 27% of the videos satisfied the PEMAT-A/V understandability threshold.

**Conclusion:**

Reliable, expert-made pro-fluoride videos attract modest audiences, whereas anti-fluoride videos can achieve a wide reach when they are easy to follow and well-structured. However, scientific accuracy alone cannot guarantee audience reach or engagement. Oral health authorities should design algorithm-sensitive, evidence-based videos that are clear and actionable.

**Innovation:**

This is the first study to integrate the PEMAT-A/V, GQS, and mDISCERN with YouTube analytics for Japanese fluoride content, thereby providing a data-driven framework for algorithm-aware oral-health messaging.

## Highlights

• Pro-fluoride videos exhibited a higher reliability score but lower audience reach.
• Understandable and well-structured anti-fluoride videos exhibited a high view rate.
• Only one-quarter of all videos satisfied the PEMAT-A/V understandability threshold.
• PEMAT-A/V, GQS, and mDISCERN triangulated with analytics reveal engagement drivers.

## 1 Introduction

In recent years, methods of obtaining health-related information have changed substantially. Although such information has traditionally been disseminated primarily through face-to-face interaction, rapid advances in digital technologies have established social media as a key health information source. Today, individuals can readily access information via computers and smartphones, enabling real-time information sharing and exchange irrespective of time or location.

These shifts in the digital landscape have significantly influenced health-related behaviors. Among various platforms, YouTube—the world's largest video-sharing service—has gained attention as an online health information source [1]. As of 2024, YouTube has approximately 2.5 billion monthly active users globally. A similar trend is evident in Japan, where a 2023 survey by the Ministry of Internal Affairs and Communications on media usage time and information behavior found that over 90% of individuals in their teens to forties use YouTube [2]. Moreover, with over 70 million monthly users, YouTube is one of Japan's most frequently used social media platforms [3].

Vlogs, or video blogs, are experience-based videos created by individuals on varied topics and are typically posted on platforms such as YouTube. Vlogs concerning medical and health issues are actively shared. For example, individuals with breast cancer [4] and gestational diabetes [5] have documented their personal experiences through publicly accessible vlogs. These videos draw viewers into their creators' everyday lives; therefore, they tend to achieve high engagement rates, with audiences often continuing to watch subsequent content [6]. Such vlog-based services significantly contribute to shaping patients' experiences in the progression and management of chronic diseases.

Nevertheless, concerns persist regarding the accuracy and reliability of health-related information on YouTube. As anyone with an account can freely upload content, a risk exists that unverified information may circulate without adequate fact-checking [7]. Considering YouTube's widespread influence and ease of access, ensuring its health-related content’ quality and accuracy is imperative.

Prior research has suggested that individuals with low health literacy are more likely to obtain health information through television, social media, blogs, or celebrity websites than their higher-literacy counterparts [8]. However, some of these sources, particularly celebrity websites, blogs, and social media, may provide lower-quality health information than professional sources, such as physicians and dentists [8].

Additionally, studies have indicated that dental caries, like other health disparities, are associated with social determinants and have been referred to as a “canary in the coalmine” [9]. Beyond platform uptake, experimental and review evidence has indicated that presentation design directly impacts comprehension and behavior. Randomized experiments manipulating processing fluency (e.g., typography and layout) have been demonstrated to increase perceived ease, self-efficacy, and behavioral intentions [10]. Meanwhile, systematic reviews have revealed that transparent visual aids reliably enhance risk understanding and informed decision-making across diverse audiences [11,12]. Consequently, ensuring the online availability of accurate, accessible, and actionable health information to all individuals is essential for addressing health disparities.

Population-based studies have suggested that comprehensive health literacy in Japan is heterogeneous and, on average, lower than that in Europe, underscoring the necessity for comprehensible and actionable materials. Validated Japanese instruments (e.g., a Japanese adaptation of the PEMAT) enable the systematic appraisal of these properties in Japanese-language materials. Combined with the characteristics of Japanese orthography (kanji–kana mixing), these factors justify a design-aware approach to evaluating and enhancing online health information in Japan.

Fluoride use is a particularly effective and widely recognized public health intervention for preventing dental caries, a preventable condition [13]. Application methods are generally classified into systemic and topical approaches [14,15]. Community-level water fluoridation is among the most extensively implemented systemic interventions. As of 2012, over 420 million individuals worldwide used fluoridated water, including approximately 50 million who relied on naturally fluoridated sources and approximately 370 million relying on water supplies adjusted to optimal or near-optimal fluoride concentrations [16]. For topical application, fluoride-containing toothpaste, mouth rinses, and professionally applied fluoride treatments in dental clinics are commonly employed at the individual level [14]. In Japan, community water fluoridation has not been implemented as a public health measure, and fluoride is primarily applied at the individual level. In Japan, local governments implement school-based fluoride mouth-rinse programs (S-FMR). Recent studies have reported that geographic variation in naturally occurring fluoride in tap water is associated with lower caries experience among children and that prefectures with broader S-FMR dissemination exhibit lower mean DMFT and smaller inter-prefectural inequalities. However, S-FMR has not been universally adopted across municipalities, and fluoride-based caries prevention remains centered on individual-level behaviors. Thus, for effective caries prevention, individuals must act based on accurate information regarding fluoride use. However, no study has evaluated the understandability and actionability of fluoride-related information readily accessible to the general public.

Fluoride has frequently become the subject of intense debate on social media platforms, where pro- and anti-fluoride perspectives often clash. Opponents of fluoride typically include general users, influencers, and activists lacking dental and scientific expertise. Prior research has revealed that a significant portion of fluoride-related online content is either negative or misleading. An analysis of widely viewed fluoride-related YouTube videos identified an anti-fluoride bias, with the content focusing more on perceived risks than on documented benefits. Among a sample of 100 such videos, most were produced by non-experts, with user-generated content accounting for approximately 69% of total views, suggesting that anti-fluoride or sensational content frequently achieves a greater reach than expert-driven content [17]. On Instagram, anti-fluoride posts often feature emotionally charged messages, such as “Fluoride is poisoning you and your children.” [18]. Posts with strongly negative or sensational tones elicit greater engagement with social media algorithms. Fear-inducing appeals and provocative statements by opponents generate more shares and comments, thereby amplifying the content's reach.

A marked difference exists in both the nature and reliability of online information about fluoride, depending on whether it is disseminated by supporters or opponents, which may influence the public understanding of the topic. A study on the readability of web pages concerning fluoride use found that anti-fluoride websites are generally written in a more accessible style than pro-fluoride websites, potentially making it easier for a wider audience to comprehend, contributing to public confusion on the issue [19]. Pro-fluoride information generally originates from expert sources, with many posts and videos produced by dentists, pediatricians, and public health organizations. However, the reach of these sources may be limited. As previously noted, consumer-generated videos on YouTube receive substantially more views than those produced by experts [17]. Similarly, an analysis of Instagram indicated that most fluoride-related posts are created by non-experts and often achieve high engagement, even when they contain misinformation.

Growing evidence has suggested that online misinformation is associated with fluoride hesitation or refusal in specific communities. For instance, dental practitioners have reported an increasing number of parents declining fluoride application for their children, citing online sources such as “I read it on Facebook” or “I saw it on YouTube” that claim fluoride is harmful [18]. In a study involving interviews with mothers of young children residing in areas with strong anti-fluoride sentiment, two-thirds reported discussing or obtaining fluoride-related information through social networks, including family, friends, other parents, and social media contacts. Numerous mothers indicated that they received conflicting messages. While some sources (e.g., pediatric dentists or scientifically inclined acquaintances) provided reassurance about fluoride's benefits, others (e.g., neighbors or Facebook groups) issued warnings. This inconsistency across multiple sources caused confusion regarding whom to trust and made it difficult for these mothers to discern which fluoride-related advice was most accurate [20]. Moreover, opposition to fluoride use has been identified as a key factor influencing parental refusal of fluoride treatment offered by dental professionals [21]. Studies have also found that parents opposing fluoride often exhibit resistance to vaccinations. Such refusals reportedly contribute to increased disease burden, rising healthcare costs, and preventable illness and suffering among children and their surrounding communities [22].

In the formation of attitudes toward health information, non-logical factors—such as the mode of presentation and perceptions of the information source—are as influential as information reliability or scientific validity. The Elaboration Likelihood Model (ELM) conceptualizes behavior change through two cognitive pathways: the central route, which involves deep processing based on content; and the peripheral route, which involves superficial processing based on emotions or impressions. This framework has been widely applied to evaluate health communication within medical and public health domains [23,24]. Furthermore, individuals tend to imitate others' behaviors or interpret them correctly when faced with uncertainty in their judgment. This psychological phenomenon, referred to as social proof, significantly influences individual decision-making. In the context of health-related topics, platforms such as YouTube function as spaces for peer-based information exchange and support among individuals with similar concerns and impact viewers' knowledge, attitudes, and behaviors.

In recent years, several standardized tools have been developed to assess health information disseminated via social media platforms, such as YouTube; prominent examples include the Patient Education Materials Assessment Tool for Audiovisual Materials (PEMAT-A/V) [25], the Global Quality Score (GQS) [26], and the modified DISCERN (mDISCERN) [27]. These tools enable the systematic evaluation of online health education content's understandability, actionability, and credibility. Previous studies employing these tools have assessed the educational quality and trustworthiness of YouTube videos on various topics, including food poisoning and COVID-19 [28], and within dentistry, on the safety of dental radiography [29] and pediatric oral hygiene instruction [30]. However, to our knowledge, no study has examined YouTube videos on the fluoride use for preventing caries with respect to comprehensibility, practical applicability, scientific reliability, or structural coherence.

Prior YouTube evaluations across telemedicine and urologic oncology have consistently reported low PEMAT A/V scores—particularly for actionability—and only fair DISCERN/GQS performance, with persistent misinformation concerns [31,32]. Similar limitations have been noted for placenta accreta and PDE5 inhibitor content, and mixed quality for prostate cancer mental health videos [[32], [33], [34]]. However, no study has examined Japanese fluoride content while integrating these quality metrics with YouTube analytics.

Previous studies have indicated that factual information alone is insufficient to persuade individuals that fluoride's benefits outweigh its risks. Furthermore, when individuals perceive fluoride as beneficial, their risk perception generally decreases [35]. Therefore, this study aims to compare the quality (understandability, actionability, reliability) and viewer engagement of pro- and anti-fluoride Japanese YouTube videos.

### 1.1 Research question

Does a difference exist in the degree of understandability, actionability, flow, comprehensiveness, and scientific reliability between pro- and anti-fluoride content shared on social media?

## 2 Materials and methods

### 2.1 Study design

We systematically and quantitatively analyzed YouTube videos on fluoride use for caries prevention using standardized health information evaluation tools.

### 2.2 Search strategy

#### 2.2.1 Video selection and eligibility

On October 15, 2023, we systematically searched YouTube for relevant videos using keyword combinations entered in Japanese text (listed in Supplementary Table 1, with English translations provided)—following methodologies used in previous research analyzing YouTube videos on health-related information [36]. These keywords were identified using Google Trends to capture terms commonly utilized by the general public; we included the top queries from the preceding 12 months to balance recency with sufficient search volume for meaningful trend identification. A comprehensive search was conducted on YouTube using a range of relevant keywords, including “fluoride,” as well as additional terms identified via Google Trends, such as “fluoride AND coating,” “fluoride AND processing,” “dentist AND fluoride,” “fluoride AND coat,” “fluoride AND toothpaste,” “fluoride AND pan coating,” “fluoride AND resin,” “fluoride AND danger,” “fluoride AND rinse,” and “fluoride.” The search results were categorized based on “view count.” The top 50 videos for each keyword were selected for analysis. The search was conducted using Google Chrome in incognito mode to prevent the influence of personal browsing history or tailored search results; the region was set to Japan. After each search, the browser was closed and reopened in incognito mode to minimize personalization. For each search formula, the top 50 results were reviewed on February 10, 2025. Only videos with clear pro- or anti-stances were included, while those categorized as “other” (*n* = 17) were excluded. The exclusion criteria were as follows: duplicate videos, videos irrelevant to the search term (such as content about Teflon-coated cookware), videos shorter than one minute, non-Japanese language videos, and videos intended for dental hygienists. Videos targeting dental hygienists were excluded because the PEMAT-A/V, GQS, and mDISCERN instruments are designed to evaluate patient-facing educational materials. Including professional-only content would fall outside the intended scope of these tools and could bias assessments of understandability and actionability for the lay public. Additionally, one video was removed because it had been deleted for violating YouTube Community Guidelines. Finally, 84 videos were included. This study analyzed publicly available online content and did not involve human subjects. Institutional review board approval was, therefore, waived. The study was conducted in accordance with institutional guidelines and PRISMA principles adapted to digital media studies. Complete search terms (Japanese and English) are provided in Supplementary Table 1, and video classification criteria are detailed in Appendix A.

### 2.3 Data extraction

#### 2.3.1 Basic characteristics

The following basic information was collected from each video: number of days since upload, total view count, number of likes, source, intended audience, and content. To assess viewership trends, the number of views was divided by the number of days since upload to calculate the average number of views per day.

We identified each video's source based on the YouTube channel that uploaded it. We categorized it into six groups: dentist/dental hygienist, local government, company, dental association, layperson, and unknown. “Company” referred to dental product manufacturers. “Unknown” was defined as videos with no clear author information. We also identified the intended audience of the videos. We judged them based on the video titles.

### 2.4 Content characteristics

The first author (HSN) assessed the intended audience for each video and classified them as either the general public or dental hygienists. Moreover, HSN examined video content to determine its stance on fluoride use for caries prevention, categorizing each video as pro, anti, or other based on its central message. To ensure systematic classification, the first author (HSN) categorized each video as pro, anti, or other. This process was guided by a predefined set of criteria (detailed in Appendix A) focusing on the video's central message, narration, and visual elements to minimize subjective judgment. Classification criteria included the video's overall stance as expressed in the narration, subtitles, and visual elements, for subsequent analyses of video characteristics and quality assessment using PEMAT-A/V, GQS, and mDISCERN.

### 2.5 Evaluation methods

#### 2.5.1 Understandability and actionability

We used the validated Japanese version of PEMAT-A/V to rate two domains—understandability (12 items) and actionability (4 items)—for each video [37]. Items were scored dichotomously (agree = 1; disagree = 0); domain scores were computed as (sum of “agree” / number of applicable items) × 100, excluding items marked not applicable. Following prior applications, we interpreted a score of ≥70% as an acceptable benchmark for both domains [37]. Appendix C concisely summarizes domains, scoring, and thresholds (full item wording in Appendix B).

#### 2.5.2 Natural flow and comprehensiveness

Each video was assessed using the GQS, a five-point Likert scale designed to evaluate the presented information's overall natural flow and comprehensiveness [26]. The GQS is widely used to evaluate health- and medicine-related video content. It comprises a single-item measure with scores ranging from 1 (poor quality) to 5 (excellent quality; Appendix D).

### 2.6 Reliability

We applied the mDISCERN items using their Japanese translation, which was adapted from the validated Japanese version of the original DISCERN [27]. The mDISCERN index was employed as a scoring system to assess the content's reliability and accuracy, using a five-point scale [27]. The score is based on five criteria: aim, presentation accuracy, balance, reference, and mentioning uncertainty. Higher total scores reflect greater overall reliability (Appendix E).

### 2.7 Statistical analysis

Two independent reviewers (HSN, a dentist; EF, a health communication professional) independently rated a random sample of 23 of 84 videos (27.4%) using the PEMAT-A/V, GQS, and mDISCERN tools. The sample was selected with a computer-generated random number table in Microsoft Excel. Agreement was assessed at the item level using Cohen's κ and Gwet's AC1, and at the scale level using intraclass correlation coefficients (ICC [1,2]; two-way random effects, absolute agreement). Discrepancies were resolved through discussion until a consensus was reached. After confirming acceptable agreement, the remaining videos were rated by HSN.

We used descriptive statistics to summarize the characteristics of the retrieved videos and calculate the PEMAT-A/V, GQS, and the mDISCERN index.

To examine attention on YouTube, we calculated each video's view rate—defined as the number of views divided by the number of days since upload (views/day). As the distribution of view rates was highly skewed, particularly among the pro-fluoride videos, which included some with exceptionally high view counts, we applied a log transformation to stabilize the variance and reduce the influence of extreme outliers. Specifically, we used the formula log(views/day +1) to avoid undefined values for zero and ensure that all values remained positive. Visual inspection also indicated that the variability (i.e., dispersion) in view rates was notably greater in the pro-fluoride group than in the anti-fluoride group. This heterogeneity makes it difficult to interpret group differences using raw values, further justifying the need for transformation. Statistical significance was defined a priori as *p* < 0.05 (two-tailed). No corrections for multiple comparisons were applied, as the analyses were exploratory. All analyses were performed in R version 4.1.1 (R Foundation for Statistical Computing). Descriptive statistics and group comparisons were conducted using base R functions. Inter-rater reliability was assessed using the ‘irr’ package (version 0.84.1) for Cohen's κ and Gwet's AC1, and the ‘psych’ package (version 2.1.9) for intraclass correlation coefficients. Spearman's rank correlations were computed using the base ‘stats’ package.

## 3 Results

**Fig. 1:**
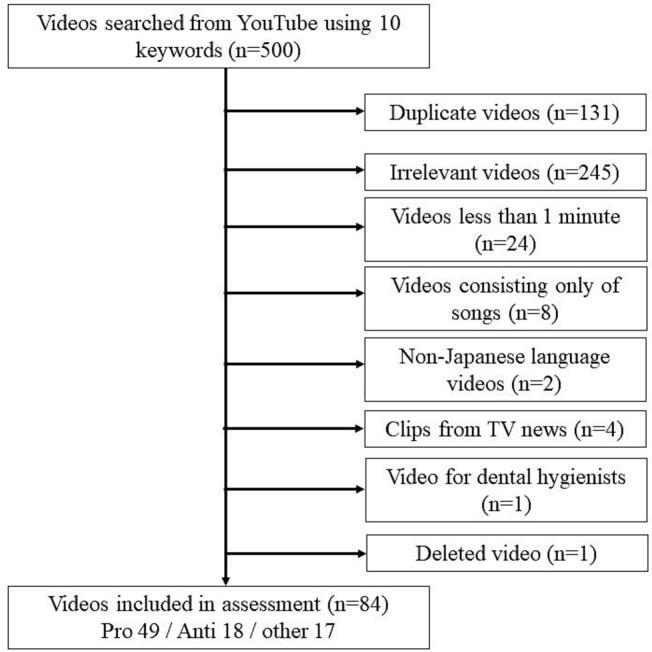
PRISMA flow diagram depicting inclusion and exclusion criteria of YouTube videos.

Fig. 1 shows the PRISMA flow of video selection. Of the 84 eligible videos, 49 were pro-fluoride, 18 were anti-fluoride, and 17 were other. As Table 1 indicates, pro-fluoride videos were mainly uploaded by dentists/dental hygienists, while most anti-fluoride videos came from laypersons, with a smaller number from unknown sources or a single dental professional. Anti-fluoride videos also received more likes on average.

**Table 1:** Video characteristics.

	Pro	Anti	Other
Basic information			
Number of videos included	49	18	17
Days since upload - maximum (days)	2648	2437	4177
Days since upload - minimum (days)	157	296	563
Days since upload - average (days)	1193	944	1465
Number of views - maximum (views)	2,475,818	283,122	4,111,810
Number of views – minimum (views)	91	658	16,731
Number of views - average (views)	151,214	76,224	588,462
Views per day - maximum	1425	327	7303
Views per day - minimum	0.05	0.45	16
Views per day - average	150	111	748
Number of likes - maximum	13,319	4312	7671
Number of likes - minimum	0	8	82
Number of likes - average	992	1524	1781
Video length - maximum (seconds)	3616	1169	1247
Video length - minimum (seconds)	66	164	79
Video length - average (seconds)	605	644	541
Video creators	Pro	Anti	Other
Dentists and dental hygienists	29	1	13
Local government	2	0	0
Companies	2	0	0
Dental association	1	0	0
Layperson	15	15	4
Unknown	0	2	0
Target audience	Pro	Anti	Other
General public	49	18	17
Dental hygienists	0	0	0

**Fig. 2:**
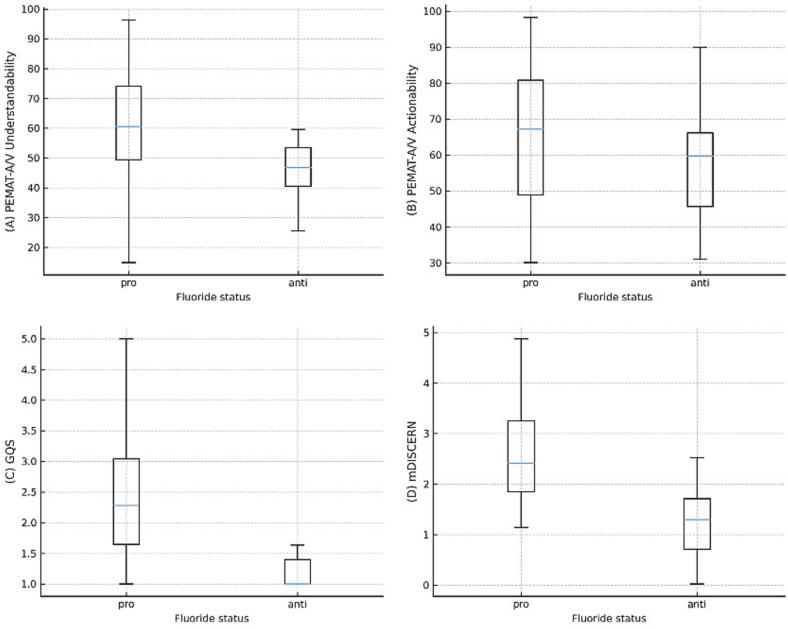
Distributions of scores by fluoride status. Boxplots show the distribution of (A) PEMAT-A/V Understandability, (B) PEMAT-A/V Actionability, (C) Global Quality Score (GQS), and (D) mDISCERN scores. Whiskers indicate the 1.5 interquartile range.

As Table 2 shows, pro-fluoride videos scored significantly higher on reliability and overall quality (mDISCERN: 2.61 ± 0.95 versus 1.29 ± 0.57, d = 1.53, 95% CI 0.89–2.17; GQS: 2.46 ± 1.36 versus 1.00 ± 0.91, d = 1.22, 95% CI 0.60–1.84; both *p* < 0.001). By contrast, PEMAT-A/V scores for understandability (58.0% versus 52.8%) and actionability (60.2% versus 53.0%) did not differ significantly between groups. Fig. 2, which presents the distributions of PEMAT-A/V, mDISCERN, and GQS scores, illustrates group medians, interquartile ranges, and outlying values for pro- versus anti-fluoride videos. However, only about one-quarter of videos satisfied the PEMAT understandability threshold, and about one in 10 fulfilled the actionability threshold, underscoring limited understandability and actionability. Among pro-fluoride sources, videos from local government and the dental association scored highest on reliability, whereas among anti-fluoride sources, videos from unknown uploaders slightly outperformed those from laypersons.

**Table 2:** Comparison of quality scores between pro- and anti-fluoride videos.⁎

Video Features	Fluoride status		
	Pro (*n* = 49)	Anti (*n* = 18)	*p*-value
PEMAT-A/V			
Understandability	58.0 ± 16.9	52.8 ± 15.8	0.226
Actionability	60.2 ± 19.6	53.0 ± 20.0	0.332
mDISCERN	2.61 ± 0.95	1.29 ± 0.57	<0.001⁎
GQS	2.46 ± 1.36	1.00 ± 0.91	<0.001⁎

GQS: Global Quality Score.

Values are presented as mean ± standard deviation.

The Mann-Whitney *U* test was used.

⁎ *p* < 0.001.

Table 3 presents correlations between content quality and engagement. Among pro-fluoride videos, no significant correlations were observed (e.g., GQS ρ = −0.24, *p* = 0.085). By contrast, among anti-fluoride videos, view rate showed a moderate to strong positive correlation with both understandability (ρ = 0.53, *p* = 0.028) and GQS (ρ = 0.53, *p* = 0.029), suggesting that clearer and better-structured anti-fluoride content tended to attract more viewers.

**Table 3:** Spearmans rank correlation (ρ) between view rate and quality scores.

	Pro (n = 49)		Anti (*n* = 18)	
	ρ	*p*-value	ρ	*p*-value
PEMAT-A/V Understandability	0.05	0.730	0.53	0.028
PEMAT-A/V Actionability	−0.10	0.470	0.41	0.100
GQS	−0.24	0.085	0.53	0.029
mDISCERN	−0.15	0.290	0.13	0.630

Appendix F presents the detailed PEMAT-A/V item-level results. Both pro- and anti-fluoride videos generally used everyday language and direct address effectively, but performed poorly on items requiring the use of tables, graphs, or diagrams. For example, in the pro-fluoride group, 87.0% of videos made their purpose completely evident from the beginning (Item 1) and 87.0% used common, everyday language (Item 3), whereas about 11% fulfilled the criterion for Item 18 (“use of tables with clear headings”). In the anti-fluoride group, 94.4% used everyday language (Item 3) and 94.4% had text on the screen that was easy to read (Item 12). Item 18 (“use of tables with clear headings”) was generally not applicable owing to the absence of tables (see Appendix F). These findings highlight the widespread lack of visual support across both groups.

Concerning inter-rater reliability, for PEMAT-A/V item ratings, Gwet's AC1 ranged from 0.48 to 0.83 (95% CIs in Appendix G); corresponding Cohen's κ values were lower, consistent with prevalence effects. At the scale level, ICCs were 0.76 for understandability and 0.51 for actionability. Appendix H reports agreement for the single-item GQS. Appendix I reports the item-level κ/AC1/ICC coefficients for mDISCERN; the global index showed moderate agreement (AC1 ≈ 0.46).

## 4 Discussion and conclusion

### 4.1 Discussion

This study evaluated 84 YouTube videos on fluoride use for caries prevention and compared pro- and anti-fluoride content using PEMAT-A/V, GQS, and mDISCERN. For PEMAT-A/V, mean understandability and actionability were slightly higher for pro-fluoride videos, but the differences were not significant. Within the pro group, videos posted by local governments and dental associations achieved particularly high mDISCERN scores, indicating stronger source credibility. By contrast, the quality of layperson-generated videos was more variable, suggesting that the creator's background influences clarity and actionability. Although prior work on webpages reported that anti-fluoride sites can be more readable than pro sites [19], direct comparison is limited because this study assessed audiovisual materials rather than text. The observed effect sizes for reliability and overall quality differences between pro- and anti-fluoride videos (Cohen's d = 1.53 for mDISCERN; d = 1.16 for GQS) represent large practical differences according to conventional interpretive benchmarks (d ≥ 0.8 indicating a large effect). These substantial effect sizes underscore that the reliability gap between expert-generated and lay-produced content is not merely statistically significant but also meaningfully large in magnitude, with important implications for public health messaging strategies.

On GQS, which reflects overall structure and coverage, and on mDISCERN, a reliability index, pro-fluoride videos significantly outperformed anti-fluoride videos, with large effect sizes indicating robust differences beyond statistical significance. Nevertheless, higher credibility and coherence did not automatically translate into greater reach. Among anti-fluoride videos, higher view counts were positively correlated with higher understandability and with higher GQS, implying that lower cognitive burden and smoother structure can drive engagement. These patterns align with the ELM: Evidentiary strength operates as a central cue, while audience engagement often relies on peripheral cues such as fluency, narrative style, and production choices. Interestingly, a notable characteristic within the pro-fluoride group was the use of vlog-style formats, particularly by laypersons. These videos, often featuring everyday clinic scenes or family routines, may not be primarily designed as expert information vehicles. However, such portrayals can shape attitudes through familiarity and empathy, potentially influencing behavior via the peripheral route [23]. This highlights a key tension: Even within the pro-fluoride camp, a divergence prevails between high-reliability content from experts and potentially more engaging, narrative-driven content from laypersons. As our findings suggest, these vlog-style formats—though effective for building empathy—may not always prioritize the high informational quality observed in expert-produced content, suggesting a potential strategy wherein health authorities could partner with trusted creators to produce expert-informed vlogs. However, such an approach necessitates careful design to leverage empathetic storytelling without trivializing the scientific information being conveyed. Even pro-fluoride videos fell short of recommended PEMAT-A/V thresholds: understandability, 56.4%; and actionability, 58.0%. Item-level patterns indicated limited use of concise tables and clear headings (PEMAT Item 18) and insufficient charts or graphs to prompt action (PEMAT Item 24). Overall, these findings underscore a persistent accuracy–engagement gap: Expert-made content tends to be more credible and coherent, yet content that is easier to process can spread more widely. Beyond the fluoride context, integrating content-quality metrics with platform analytics offers a replicable framework to study how accuracy, accessibility, and reach interact across digital health topics. Our findings align with reports from other medical domains. Studies on telemedicine and urologic immunotherapy have documented low understandability and especially low actionability on PEMAT-A/V, with only fair DISCERN and modest GQS scores, alongside persistent misinformation concerns [31,32]. Similar quality limitations have been observed for placenta accreta and PDE5 Inhibitor content, and prostate cancer mental health videos have yielded mixed quality despite covering high-need topics [32]. Collectively, these benchmarks suggest a recurring configuration in digital health: Expert-generated content generally achieves higher reliability, whereas materials that are easier to process—regardless of accuracy—typically gain greater reach. Our fluoride-related results align with this broader trend, reinforcing the need to pair evidence-rich central cues with fluency-oriented design features. Looking forward, our integrated framework, which combines PEMAT-A/V, GQS, and mDISCERN metrics with engagement analytics, may inform the development of algorithmic or semi-automated systems for scalable health content quality assessment. Future research could explore whether specific linguistic features, visual elements, or structural characteristics that predict high PEMAT-A/V or mDISCERN scores can be computationally detected through natural language processing or computer vision techniques. Such automated quality signals could potentially be incorporated into platform recommendation algorithms to promote evidence-based content more effectively, enabling real-time monitoring of health misinformation across large video corpora.

Exposure to online misinformation can cause parents to decline fluoride application for their children, and anti-fluoride beliefs have been associated with broader patterns of treatment refusal and vaccine hesitancy. These dynamics can elevate disease burden among children and communities, contribute to preventable suffering, and raise healthcare costs [22]. Addressing misinformation on widely used platforms is, therefore, a public health priority. Cultural and linguistic factors likely shape engagement with fluoride content in Japan. Population-based studies have indicated that comprehensive health literacy in Japan is lower than in Europe and heterogeneous across adults, underscoring the need to tailor materials for comprehension and action. Additionally, a validated Japanese version of the PEMAT demonstrates acceptable reliability and construct validity, supporting the use of understandability and actionability benchmarks in Japanese-language materials. Beyond measurement, randomized experiments manipulating processing fluency (e.g., typography, layout) have demonstrated that easier-to-read health materials increase perceived ease, self-efficacy, and behavioral intentions, suggesting that design choices directly enhance motivation to act. [10] Complementary evidence from systematic reviews has suggested that transparent visual aids reliably improve risk understanding and informed decision making across diverse audiences, including those with lower skills [11]. In sum, aligning credible, evidence-based messages with fluency-oriented design (captions, chaptering, concise graphics) is a plausible strategy to maximize both accessibility and persuasive impact in Japanese video health communication.

Dental caries has been described as a “canary in the coalmine” because of its close associations with socioeconomic determinants [9]. Individuals with lower health literacy are more likely to rely on potentially inaccurate sources of health information [8]. Ensuring that YouTube content about fluoride is accurate, comprehensible, and actionable is, thus, essential to mitigate disparities and reduce the disproportionate impact of oral disease on vulnerable populations.

Trusted sources—particularly local governments and dental associations—should continue to maintain high GQS and mDISCERN performance while systematically improving understandability and actionability. Design strategies that reduce cognitive load and guide concrete behaviors can bridge the accuracy–engagement divide. Examples include chaptered segments, concise infographics, and clear subtitles to reinforce narration, as well as step-by-step demonstrations of product selection, appropriate dosage, and use frequency to support behavior change [23]. Aligning central-route content (evidence and guidance) with peripheral-route supports (fluency, narrative structure, visual scaffolds) may increase both reach and sustained persuasion among audiences with diverse health literacy levels.

#### 4.1.1 Limitations

This study had several limitations. First, video sampling was conducted based on specific search terms and search dates and, hence, did not represent the entirety of the fluoride-related content on YouTube. Sorting by total view count likely over-represented highly popular videos while under-representing less-viewed yet potentially influential content. Furthermore, our sample of 84 videos—though systematically selected—might not have fully captured the complete breadth of fluoride-related content on the platform. Second, because YouTube is dynamic, our findings should be interpreted as time-bounded; moreover, the interval between the initial search (October 15, 2023) and the focused review of top results (February 10, 2025) may have exposed the dataset to changes in ranking dynamics. This rapid evolution of content suggests that one-off analyses are insufficient; continuous monitoring of the information ecosystem and potential collaborations with platforms to promote reliable content may be necessary to mitigate the spread of misinformation. Third, we did not model YouTube's recommendation algorithms, which strongly influence visibility and engagement. Fourth, although standardized tools were used, rater subjectivity cannot be completely excluded. Inter-rater agreement for GQS (a single-item global rating) and for the mDISCERN index was moderate, which tempers the strength of inferences drawn from these scales; results were interpreted cautiously. Fifth, viewer characteristics (e.g., age, health literacy, prior attitudes) were not assessed, limiting generalizability across subgroups. Finally, analyses were restricted to Japanese-language videos and were observational in nature; causal inferences regarding how video features drive engagement cannot be drawn. Future research should involve more detailed qualitative analyses examining how pro- and anti-fluoride content influences viewer attitudes and behaviors.

### 4.2 Innovation

This is the first study to integrate the PEMAT-A/V, GQS, and mDISCERN with YouTube analytics for Japanese fluoride content. By integrating established quality metrics with platform data, it provides a data-driven framework to inform algorithm-aware oral-health communication strategies.

### 4.3 Conclusion

This study evaluated the quality of pro- and anti-fluoride YouTube videos on fluoride use, using the PEMAT-A/V, GQS, and mDISCERN to assess their understandability, actionability, natural flow and comprehensiveness, and reliability. Although pro-fluoride videos were rated highly for structure and reliability, they did not necessarily attract many views. By contrast, some anti-fluoride videos containing misinformation received more views when they scored well in terms of understandability and natural flow.

The dissemination of health information must prioritize not only scientific accuracy but also strategic communication approaches that convey evidence-based content in ways that are accessible and actionable for diverse audiences. To translate these findings into practice, public health authorities and professional dental organizations should invest in communication strategies that are not only evidence-based but also algorithm-sensitive. In short term, collaborations with digital platforms (e.g., YouTube) may help increase the visibility of reliable videos. In the long term, capacity building efforts, such as training health communication specialists, are needed to develop engaging, audience resonant storytelling while maintaining scientific accuracy. Such measures could enhance trustworthy oral health information's visibility and impact, thereby counteracting misinformation and supporting equitable disease prevention at the population level. Future work could also extend this approach to AI-assisted screening, for example using natural language processing or computer vision to detect features associated with higher quality content at scale. Ultimately, the integrated methodological framework employed herein—particularly the combination of validated quality metrics with platform analytics—offers a replicable model for future research to assess and enhance health communication across a broad range of topics on digital platforms.

## CRediT authorship contribution statement

**Hikari Sophia Nagao:** Visualization, Investigation, Writing – review & editing, Writing – original draft. **Tsuyoshi Okuhara:** Validation, Supervision, Project administration. **Emi Furukawa:** Validation, Writing – review & editing. **Hiroko Okada:** Supervision, Writing – review & editing. **Takahiro Kiuchi:** Supervision.

## Funding sources

This work was supported by the 10.13039/501100001691Japan Society for the Promotion of Science KAKENHI (25K13483).

## Declaration of competing interest

The authors declare that they have no known competing financial interests or personal relationships that could have appeared to influence the work reported in this paper.

## Figures and Tables

**Table:** 

Pros	The video concludes that fluoride use is advisable. Even in the absence of a clear conclusion, the content evidently advocates for fluoride use.
Cons	The video concludes that fluoride use is inadvisable. Even in the absence of a clear conclusion, the content evidently opposes fluoride use or asserts its lack of necessity.
Other	The video presents both pro- and anti-fluoride viewpoints (e.g., highlighting both benefits and risks, or suggesting that fluoride is necessary for children but not for adults).

**Table:** 

Item #	Item	Response Options
UNDERSTANDABILITY		
TOPIC: CONTENT		
1	The material makes its purpose completely evident from the beginning.	Disagree = 0, Agree = 1
TOPIC: WORD CHOICE & STYLE		
3	The material uses common, everyday language.	Disagree = 0, Agree = 1
4	When used, medical terms are defined.	Disagree = 0, Agree = 1
TOPIC: ORGANIZATION		
7	The material breaks or “chunks” information into short sections.	Disagree = 0, Agree = 1, Very short material* = N/A
8	The material's sections have informative headers.	Disagree = 0, Agree = 1, Very short material* = N/A
9	The material presents information in a logical sequence.	Disagree = 0, Agree = 1
10	The material provides a summary.	Disagree = 0, Agree = 1, Very short material* = N/A
TOPIC: LAYOUT & DESIGN		
11	The material uses visual cues (e.g., arrows, boxes, bullets, bold, larger font, highlighting) to draw attention to key points.	Disagree = 0, Agree = 1 Video = N/A
12	Text on the screen is easy to read.	Disagree = 0, Agree = 1, No text or all text is narrated = N/A
13	The material allows the user to hear the words clearly.	Disagree = 0, Agree = 1, No narration = N/A
TOPIC: USE OF VISUAL AIDS		
17	The material uses illustrations and photographs that are clear and uncluttered.	Disagree = 0, Agree = 1, No visual aids = N/A
18	The material uses simple tables with short and clear row and column headings.	Disagree = 0, Agree = 1, No tables = N/A
ACTIONABILITY		
19	The material clearly identifies at least one action the user can take.	Disagree = 0, Agree = 1
20	The material addresses the user directly when describing actions.	Disagree = 0, Agree = 1
21	The material breaks down any action into explicit steps.	Disagree = 0, Agree = 1
24	The material explains how to use the charts, graphs, tables, or diagrams to take actions.	Disagree = 0, Agree = 1, No charts, graphs, tables, or diagrams = N/A

**Table:** 

Domain	Items (n)	What it captures (examples)	Scoring per item	Domain score (0−100)	Adequacy threshold
Understandability	12	Clarity, plain language, organization, visual support (e.g., headings, graphics)	Agree = 1; Disagree = 0	(Sum of agrees ÷ applicable items) × 100	≥ 70%
Actionability	4	Presence of specific steps, cues to action, examples showing “what to do”	Agree = 1; Disagree = 0	(Sum of agrees ÷ applicable items) × 100	≥ 70%
Items marked “not applicable” are excluded from the denominator. See Appendix 2 for the complete item list and examples.					

**Table:** 

Score	Global Score Description
1	Poor quality, poor flow of the site, most information missing, not at all useful for patients
2	Generally poor quality and poor flow, some information listed but many important topics missing, of very limited use to patients
3	Moderate quality, suboptimal flow, some important information is adequately discussed but others poorly discussed, somewhat useful for patients
4	Good quality and generally good flow, most of the relevant information is listed, but some topics not covered, useful for patients
5	Excellent quality and excellent flow, very useful for patients

**Table:** 

1	Is the aim clear, concise, understandable?
2	Are sources of information reliable?
3	Is the information presented balanced and unbiased?
4	Are additional sources of information listed?
5	Does the video address areas of uncertainty?

**Table:** 

Item #	Item	Item score			
		Pro (n = 49)		Anti (n = 18)	
		mean	SD	mean	SD
UNDERSTANDABILITY		58.03	16.77	52.80	15.78
TOPIC: CONTENT					
1	The material makes its purpose completely evident from the beginning.	0.87	0.34	0.65	0.48
TOPIC: WORD CHOICE & STYLE					
3	The material uses common, everyday language.	0.87	0.34	0.94	0.24
4	When used, medical terms are defined.	0.30	0.46	0.35	0.48
TOPIC: ORGANIZATION					
7	The material breaks or “chunks” information into short sections.	0.56	0.50	0.41	0.49
8	The material's sections have informative headers.	0.28	0.45	0.29	0.46
9	The material presents information in a logical sequence.	0.83	0.37	0.24	0.42
10	The material provides a summary.	0.15	0.36	0.29	0.46
TOPIC: LAYOUT & DESIGN					
11	The material uses visual cues (e.g., arrows, boxes, bullets, bold, larger font, highlighting) to draw attention to key points.	0.78	0.42	0.76	0.42
12	Text on the screen is easy to read.	0.94	0.24	0.94	0.24
13	The material allows the user to hear the words clearly.	0.93	0.25	0.87	0.34
TOPIC: USE OF VISUAL AIDS					
17	The material uses illustrations and photographs that are clear and uncluttered.	0.82	0.38	0.77	0.42
18	The material uses simple tables with short and clear row and column headings.	0.11	0.31	N/A	N/A
ACTIONABILITY		60.20	19.44	52.95	20.02
19	The material clearly identifies at least one action the user can take.	0.74	0.44	0.76	0.42
20	The material addresses the user directly when describing actions.	0.74	0.44	0.82	0.38
21	The material breaks down any action into explicit steps.	0.17	0.37	0.00	0.00
24	The material explains how to use the charts, graphs, tables, or diagrams to take actions.	0.00	0.00	N/A	N/A

**Table:** 

		Score Results (sum of two raters)						Inter-rater reliability									
Item #	Item	1	(%)	0	(%)	N/A	(%)	% Agree	Cohen's κ	(95% CI)		Gwet's AC1	(95% CI)		ICC	(95% CI)	
**UNDERSTANDABILITY**															**0.76**	**(0.67, 0.84)**	
TOPIC: CONTENT						–	–										
1	The material makes its purpose completely evident from the beginning.	35	76.1	11	23.9	–	–	91.3	0.49	(0.00, 0.82)		0.81	(0.59, 0.94)				
TOPIC: WORD CHOICE & STYLE																	
3	The material uses common, everyday language.	31	67.4	15	32.6	–	–	91.3	0.66	(0.33, 0.91)		0.83	(0.63, 0.95)				
4	When used, medical terms are clearly defined.	16	34.8	30	65.2	–	–	73.9	0.48	(0.20, 0.73)		0.48	(0.20, 0.73)				
TOPIC: ORGANIZATION																	
7	The material breaks or “chunks” information into short sections.	30	65.2	16	34.8	0	0.0	82.6	0.69	(0.44, 0.89)		0.69	(0.44, 0.89)				
8	The material's sections have informative headers.	25	54.3	21	45.7	0	0.0	87.0	0.78	(0.57, 0.95)		0.78	(0.57, 0.95)				
9	The material presents information in a logical sequence.	36	78.3	10	21.7	–	–	78.3	0.54	(0.26, 0.78)		0.54	(0.27, 0.78)				
10	The material provides a summary.	10	21.7	35	76.1	1	2.1	82.6	0.65	(0.38, 0.87)		0.65	(0.38, 0.87)				
TOPIC: LAYOUT & DESIGN																	
11	The material uses visual cues (e.g., arrows, boxes, bullets, bold, larger font, highlighting) to draw attention to key points.	38	82.6	8	17.4	–	–	87.0	0.73	(0.49, 0.92)		0.73	(0.49, 0.92)				
12	Text on the screen is easy to read.	36	78.2	8	17.4	2	4.3	82.6	0.35	(−0.04, 0.70)		0.67	(0.40, 0.87)				
13	The material allows the user to hear the words clearly.	31	67.4	13	28.3	2	4.3	87.0	0.72	(0.47, 0.92)		0.72	(0.48, 0.92)				
TOPIC: USE OF VISUAL AIDS																	
17	The material uses illustrations and photographs that are clear and uncluttered.	23	50.0	16	34.8	7	15.2	78.3	0.55	(0.29, 0.79)		0.56	(0.29, 0.79)				
18	The material uses simple tables with short and clear row and column headings.	3	6.5	4	8.7	39	84.8	82.6	0.65	(0.38, 0.87)		0.65	(0.38, 0.87)				
**ACTIONABILITY**															**0.51**	**(0.42, 0.59)**	
19	The material clearly identifies at least one action the user can take.	43	93.5	3	6.5	–	–	87.0	0.35	(−0.13, 0.72)		0.76	(0.51, 0.93)				
20	The material addresses the user directly when describing actions.	41	89.1	5	10.9	–	–	82.6	0.69	(0.44, 0.90)		0.69	(0.44, 0.90)				
21	The material breaks down any action into explicit steps.	9	19.6	37	80.4	–	–	91.3	0.40	(−0.19, 0.80)		0.81	(0.53, 0.96)				
24	The material explains how to use the charts, graphs, tables, or diagrams to take actions.	2	4.3	8	17.4	36	78.3	82.6	0.65	(0.38, 0.87)		0.65	(0.38, 0.87)				

**Table:** 

	% Agree	Cohen's κ	(95% CI)	Gwet's AC1	(95% CI)	ICC	(95% CI)
Global Quality Score	56.5%	0.45	(0.177, 0.704)	0.46	(0.191, 0.712)	0.79	(0.579, 0.908)

**Table:** 

		Score Results (sum of two raters)									
Item #	Item	1	(%)	0	(%)	Cohen's κ	(95% CI)	Gwet's AC1	(95% CI)	ICC	(95% CI)
1	Is the aim clear, concise, understandable?	27	58.7	19	41.3	0.56	(0.22, 0.90)	0.58	(0.24, 0.91)	0.57	(0.22, 0.91)
2	Are sources of information reliable?	17	37.0	29	63.0	0.73	(0.41, 1.00)	0.76	(0.49, 1.00)	0.73	(0.44, 1.00)
3	Is the information presented balanced and unbiased?	18	39.1	28	60.9	0.46	(0.05, 0.82)	0.50	(0.13, 0.85)	0.47	(0.06, 0.82)
4	Are additional sources of information listed?	16	34.8	30	65.2	0.43	(0.02, 0.78)	0.52	(0.14, 0.84)	0.44	(0.02, 0.80)
5	Does the video address areas of uncertainty?	16	34.8	30	65.2	0.47	(0.18, 0.80)	0.52	(0.17, 0.86)	0.48	(0.15, 0.82)

## Appendix B PEMAT-A/V items

* A very short audiovisual material is defined as a video or multimedia presentation that is under 1 min, or a multimedia material that has 6 or fewer slides or screenshots.

## Appendix F The mean score for each of the PEMAT-A/V items

* PEMAT items were scored with a binary scale (agree = 1 or disagree = 0).

Scores were calculated as calculated as percentages of total possible points, excluding not applicable items.

## Appendix G Item-level inter-rater reliability results for items in the Japanese PEMAT-A/V

We used κ coefficients to evaluate each item rather than the total score. For Understandability and Actionability scores, we calculated Cohen's κ and the intra-class coefficient(ICC).

## Appendix J Supplementary data

Supplementary material 1 Image 1

Supplementary material 2 Image 2

Supplementary material 3 Image 3

Supplementary material 4 Image 4

Supplementary material 5 Image 5

Supplementary material 6 Image 6

Supplementary material 7 Image 7

Supplementary material 8 Image 8

Supplementary material 9 Image 9

Supplementary material 10 Image 10
