# Supplementary material for: Evaluating fluoride-related YouTube videos in Japan: A comparative analysis of understandability, actionability, and reliability between pro- and anti-fluoride content
Source: PEC Innov. 2026 Feb 8;8:100458. doi: 10.1016/j.pecinn.2026.100458 (PMC12914852; doi:10.1016/j.pecinn.2026.100458)
Supplement: Supplementary file 1 — Supplementary material 1 [file mmc1.docx]

| **Supplementary Table 1. Japanese keyword combinations used in the YouTube search** | |
| --- | --- |
| Japanese | English |
| フッ素　コーティング | fluoride AND coating |
| フッ素　加工 | fluoride AND processing |
| 歯医者　フッ素 | dentist AND fluoride |
| フッ素　コート | fluoride AND coat |
| フッ素　歯磨き粉 | fluoride AND toothpaste |
| フッ素　加工　フライパン | fluoride AND pan coating |
| フッ素　樹脂 | fluoride AND resin |
| フッ素　危険 | fluoride AND danger |
| フッ素　うがい | fluoride AND rinse |
| ふっそ | fluoride |
