# Supplementary material for: Evaluating fluoride-related YouTube videos in Japan: A comparative analysis of understandability, actionability, and reliability between pro- and anti-fluoride content
Source: PEC Innov. 2026 Feb 8;8:100458. doi: 10.1016/j.pecinn.2026.100458 (PMC12914852; doi:10.1016/j.pecinn.2026.100458)
Supplement: Supplementary file 3 — Supplementary material 3 [file mmc3.docx]

| **Appendix 2. PEMAT-A/V items** | |  |  |  |  |  |
| --- | --- | --- | --- | --- | --- | --- |
|  |  |  |  |  |  |  |
| Item # | Item | Response Options |  |  |  |  |
| UNDERSTANDABILITY | |  |  |  |  |  |
| TOPIC: CONTENT | |  |  |  |  |  |
| 1 | The material makes its purpose completely evident from the beginning. | Disagree=0, Agree=1 |  |  |  |  |
| TOPIC: WORD CHOICE & STYLE | |  |  |  |  |  |
| 3 | The material uses common, everyday language. | Disagree=0, Agree=1 |  |  |  |  |
| 4 | When used, medical terms are defined. | Disagree=0, Agree=1 |  |  |  |  |
| TOPIC: ORGANIZATION | |  |  |  |  |  |
| 7 | The material breaks or “chunks” information into short sections. | Disagree=0, Agree=1, Very short material*=N/A |  |  |  |  |
| 8 | The material’s sections have informative headers. | Disagree=0, Agree=1, Very short material*=N/A |  |  |  |  |
| 9 | The material presents information in a logical sequence. | Disagree=0, Agree=1 |  |  |  |  |
| 10 | The material provides a summary. | Disagree=0, Agree=1, Very short material*=N/A |  |  |  |  |
| TOPIC: LAYOUT & DESIGN | |  |  |  |  |  |
| 11 | The material uses visual cues (e.g., arrows, boxes, bullets, bold, larger font, highlighting) to draw attention to key points. | Disagree=0, Agree=1 Video=N/A |  |  |  |  |
| 12 | Text on the screen is easy to read. | Disagree=0, Agree=1, No text or all text is narrated=N/A |  |  |  |  |
| 13 | The material allows the user to hear the words clearly. | Disagree=0, Agree=1, No narration=N/A |  |  |  |  |
| TOPIC: USE OF VISUAL AIDS | |  |  |  |  |  |
| 17 | The material uses illustrations and photographs that are clear and uncluttered. | Disagree=0, Agree=1, No visual aids=N/A |  |  |  |  |
| 18 | The material uses simple tables with short and clear row and column headings. | Disagree=0, Agree=1, No tables=N/A |  |  |  |  |
| ACTIONABILITY | |  |  |  |  |  |
| 19 | The material clearly identifies at least one action the user can take. | Disagree=0, Agree=1 |  |  |  |  |
| 20 | The material addresses the user directly when describing actions. | Disagree=0, Agree=1 |  |  |  |  |
| 21 | The material breaks down any action into explicit steps. | Disagree=0, Agree=1 |  |  |  |  |
| 24 | The material explains how to use the charts, graphs, tables, or diagrams to take actions. | Disagree=0, Agree=1, No charts, graphs, tables, or diagrams=N/A |  |  |  |  |
| * A very short audiovisual material is defined as a video or multimedia presentation that is under 1 minute, or a multimedia material that has 6 or fewer slides or screenshots. | | | | | | |
