# Supplementary material for: Evaluating fluoride-related YouTube videos in Japan: A comparative analysis of understandability, actionability, and reliability between pro- and anti-fluoride content
Source: PEC Innov. 2026 Feb 8;8:100458. doi: 10.1016/j.pecinn.2026.100458 (PMC12914852; doi:10.1016/j.pecinn.2026.100458)
Supplement: Supplementary file 4 — Supplementary material 4 [file mmc4.docx]

| **Appendix 3. PEMAT-A/V summary (domains, items, scoring, and thresholds)** | | |  |  |  |
| --- | --- | --- | --- | --- | --- |
| **Domain** | **Items (n)** | **What it captures (examples)** | **Scoring per item** | **Domain score (0–100)** | **Adequacy threshold** |
| Understandability | 12 | Clarity, plain language, organization, visual support (e.g., headings, graphics) | Agree = 1; Disagree = 0 | (Sum of agrees ÷ applicable items)×100 | ≥ 70% |
| Actionability | 4 | Presence of specific steps, cues to action, examples showing “what to do” | Agree = 1; Disagree = 0 | (Sum of agrees ÷ applicable items)×100 | ≥ 70% |
| Items marked “not applicable” are excluded from the denominator. See Appendix 2 for the complete item list and examples. | | | |  |  |
