# Supplementary material for: Evaluating fluoride-related YouTube videos in Japan: A comparative analysis of understandability, actionability, and reliability between pro- and anti-fluoride content
Source: PEC Innov. 2026 Feb 8;8:100458. doi: 10.1016/j.pecinn.2026.100458 (PMC12914852; doi:10.1016/j.pecinn.2026.100458)
Supplement: Supplementary file 5 — Supplementary material 5 [file mmc5.docx]

| **Appendix 4. Global Quality Scale (GQS)** | |
| --- | --- |
| Score | Global Score Description |
| 1 | Poor quality, poor flow of the site, most information missing, not at all useful for patients |
| 2 | Generally poor quality and poor flow, some information listed but many important topics missing, of very limited use to patients |
| 3 | Moderate quality, suboptimal flow, some important information is adequately discussed but others poorly discussed, somewhat useful for patients |
| 4 | Good quality and generally good flow, most of the relevant information is listed, but some topics not covered, useful for patients |
| 5 | Excellent quality and excellent flow, very useful for patients |
