# Supplementary material for: Evaluating fluoride-related YouTube videos in Japan: A comparative analysis of understandability, actionability, and reliability between pro- and anti-fluoride content
Source: PEC Innov. 2026 Feb 8;8:100458. doi: 10.1016/j.pecinn.2026.100458 (PMC12914852; doi:10.1016/j.pecinn.2026.100458)
Supplement: Supplementary file 6 — Supplementary material 6 [file mmc6.docx]

| **Appendix 5.** **Modified DISCERN (mDISCERN)** | |
| --- | --- |
| 1 | Is the aim clear, concise, understandable? |
| 2 | Are sources of information reliable? |
| 3 | Is the information presented balanced and unbiased? |
| 4 | Are additional sources of information listed? |
| 5 | Does the video address areas of uncertainty? |
