# Supplementary material for: Evaluating fluoride-related YouTube videos in Japan: A comparative analysis of understandability, actionability, and reliability between pro- and anti-fluoride content
Source: PEC Innov. 2026 Feb 8;8:100458. doi: 10.1016/j.pecinn.2026.100458 (PMC12914852; doi:10.1016/j.pecinn.2026.100458)
Supplement: Supplementary file 7 — Supplementary material 7 [file mmc7.docx]

| **Appendix 6. The mean score for each of the PEMAT-A/V items** | | | | | |
| --- | --- | --- | --- | --- | --- |
|  |  |  |  |  |  |
| Item # | Item | Item score | | | |
|  |  | Pro (n = 49) | | Anti (n = 18) | |
|  |  | mean | SD | mean | SD |
| UNDERSTANDABILITY | | 58.0 | 16.8 | 52.8 | 15.8 |
| TOPIC: CONTENT | |  |  |  |  |
| 1 | The material makes its purpose completely evident from the beginning. | 0.87 | 0.34 | 0.65 | 0.48 |
| TOPIC: WORD CHOICE & STYLE | |  |  |  |  |
| 3 | The material uses common, everyday language. | 0.87 | 0.34 | 0.94 | 0.24 |
| 4 | When used, medical terms are defined. | 0.30 | 0.46 | 0.35 | 0.48 |
| TOPIC: ORGANIZATION | |  |  |  |  |
| 7 | The material breaks or “chunks” information into short sections. | 0.56 | 0.50 | 0.41 | 0.49 |
| 8 | The material’s sections have informative headers. | 0.28 | 0.45 | 0.29 | 0.46 |
| 9 | The material presents information in a logical sequence. | 0.83 | 0.37 | 0.24 | 0.42 |
| 10 | The material provides a summary. | 0.15 | 0.36 | 0.29 | 0.46 |
| TOPIC: LAYOUT & DESIGN | |  |  |  |  |
| 11 | The material uses visual cues (e.g., arrows, boxes, bullets, bold, larger font, highlighting) to draw attention to key points. | 0.78 | 0.42 | 0.76 | 0.42 |
| 12 | Text on the screen is easy to read. | 0.94 | 0.24 | 0.94 | 0.24 |
| 13 | The material allows the user to hear the words clearly. | 0.93 | 0.25 | 0.87 | 0.34 |
| TOPIC: USE OF VISUAL AIDS | |  |  |  |  |
| 17 | The material uses illustrations and photographs that are clear and uncluttered. | 0.82 | 0.38 | 0.77 | 0.42 |
| 18 | The material uses simple tables with short and clear row and column headings. | 0.11 | 0.31 | N/A | N/A |
| ACTIONABILITY | | 60.2 | 19.4 | 53.0 | 20.0 |
| 19 | The material clearly identifies at least one action the user can take. | 0.74 | 0.44 | 0.76 | 0.42 |
| 20 | The material addresses the user directly when describing actions. | 0.74 | 0.44 | 0.82 | 0.38 |
| 21 | The material breaks down any action into explicit steps. | 0.17 | 0.37 | 0.00 | 0.00 |
| 24 | The material explains how to use the charts, graphs, tables, or diagrams to take actions. | 0.00 | 0.00 | N/A | N/A |
| * PEMAT items were scored with a binary scale (agree = 1 or disagree = 0).  Domain scores were calculated as percentages of total possible points, excluding not applicable items. | | | | | |
