# Supplementary material for: Evaluating fluoride-related YouTube videos in Japan: A comparative analysis of understandability, actionability, and reliability between pro- and anti-fluoride content
Source: PEC Innov. 2026 Feb 8;8:100458. doi: 10.1016/j.pecinn.2026.100458 (PMC12914852; doi:10.1016/j.pecinn.2026.100458)
Supplement: Supplementary file 8 — Supplementary material 8 [file mmc8.docx]

| **Appendix 7.** Item-level inter-rater reliability results for items in the Japanese PEMAT-A/V | | | | | | | | |  |  |  |  |  |  |  |  |  |  |
| --- | --- | --- | --- | --- | --- | --- | --- | --- | --- | --- | --- | --- | --- | --- | --- | --- | --- | --- |
|  |  | Score Results (sum of two raters) | | | | | |  | Inter-rater reliability | | | | | | |  |  |  |
| Item # | Item | 1 | (%) | 0 | (%) | N/A | (%) |  | % Agree | Cohen’s κ | (95% CI) | | Gwet’s AC1 | (95% CI) | | ICC | (95% CI) | |
| **UNDERSTANDABILITY** | |  |  |  |  |  |  |  |  |  |  |  |  |  |  | **0.76** | **(0.67, 0.84)** | |
| TOPIC: CONTENT | |  |  |  |  | ‐ | ‐ |  |  |  |  |  |  |  |  |  |  |  |
| 1 | The material makes its purpose completely evident from the beginning. | 35 | 76.1 | 11 | 23.9 | ‐ | ‐ |  | 91.3 | 0.49 | (0.00, 0.82) | | 0.81 | (0.59, 0.94) | |  |  |  |
| TOPIC: WORD CHOICE & STYLE | |  |  |  |  |  |  |  |  |  |  |  |  |  |  |  |  |  |
| 3 | The material uses common, everyday language. | 31 | 67.4 | 15 | 32.6 | ‐ | ‐ |  | 91.3 | 0.66 | (0.33, 0.91) | | 0.83 | (0.63, 0.95) | |  |  |  |
| 4 | When used, medical terms are clearly defined. | 16 | 34.8 | 30 | 65.2 | ‐ | ‐ |  | 73.9 | 0.48 | (0.20, 0.73) | | 0.48 | (0.20, 0.73) | |  |  |  |
| TOPIC: ORGANIZATION | |  |  |  |  |  |  |  |  |  |  |  |  |  |  |  |  |  |
| 7 | The material breaks or “chunks” information into short sections. | 30 | 65.2 | 16 | 34.8 | 0 | 0.0 |  | 82.6 | 0.69 | (0.44, 0.89) | | 0.69 | (0.44, 0.89) | |  |  |  |
| 8 | The material’s sections have informative headers. | 25 | 54.3 | 21 | 45.7 | 0 | 0.0 |  | 87.0 | 0.78 | (0.57, 0.95) | | 0.78 | (0.57, 0.95) | |  |  |  |
| 9 | The material presents information in a logical sequence. | 36 | 78.3 | 10 | 21.7 | ‐ | ‐ |  | 78.3 | 0.54 | (0.26, 0.78) | | 0.54 | (0.27, 0.78) | |  |  |  |
| 10 | The material provides a summary. | 10 | 21.7 | 35 | 76.1 | 1 | 2.17 |  | 82.6 | 0.65 | (0.38, 0.87) | | 0.65 | (0.38, 0.87) | |  |  |  |
| TOPIC: LAYOUT & DESIGN | |  |  |  |  |  |  |  |  |  |  |  |  |  |  |  |  |  |
| 11 | The material uses visual cues (e.g., arrows, boxes, bullets, bold, larger font, highlighting) to draw attention to key points. | 38 | 82.6 | 8 | 17.4 | ‐ | ‐ |  | 87.0 | 0.73 | (0.49, 0.92) | | 0.73 | (0.49, 0.92) | |  |  |  |
| 12 | Text on the screen is easy to read. | 36 | 78.2 | 8 | 17.4 | 2 | 4.34 |  | 82.6 | 0.35 | (-0.04, 0.70) | | 0.67 | (0.40, 0.87) | |  |  |  |
| 13 | The material allows the user to hear the words clearly. | 31 | 67.4 | 13 | 28.3 | 2 | 4.34 |  | 87.0 | 0.72 | (0.47, 0.92) | | 0.72 | (0.48, 0.92) | |  |  |  |
| TOPIC: USE OF VISUAL AIDS | |  |  |  |  |  |  |  |  |  |  |  |  |  |  |  |  |  |
| 17 | The material uses illustrations and photographs that are clear and uncluttered. | 23 | 50.0 | 16 | 34.8 | 7 | 15.2 |  | 78.3 | 0.55 | (0.29, 0.79) | | 0.56 | (0.29, 0.79) | |  |  |  |
| 18 | The material uses simple tables with short and clear row and column headings. | 3 | 6.52 | 4 | 8.69 | 39 | 84.8 |  | 82.6 | 0.65 | (0.38, 0.87) | | 0.65 | (0.38, 0.87) | |  |  |  |
| **ACTIONABILITY** | |  |  |  |  |  |  |  |  |  |  |  |  |  |  | **0.51** | **(0.42, 0.59)** | |
| 19 | The material clearly identifies at least one action the user can take. | 43 | 93.5 | 3 | 6.52 | ‐ | ‐ |  | 87.0 | 0.35 | (-0.13, 0.72) | | 0.76 | (0.51, 0.93) | |  |  |  |
| 20 | The material addresses the user directly when describing actions. | 41 | 89.1 | 5 | 10.9 | ‐ | ‐ |  | 82.6 | 0.69 | (0.44, 0.90) | | 0.69 | (0.44, 0.90) | |  |  |  |
| 21 | The material breaks down any action into explicit steps. | 9 | 19.6 | 37 | 80.4 | ‐ | ‐ |  | 91.3 | 0.40 | (-0.19, 0.80) | | 0.81 | (0.53, 0.96) | |  |  |  |
| 24 | The material explains how to use the charts, graphs, tables, or diagrams to take actions. | 2 | 4.3 | 8 | 17.4 | 36 | 78.3 |  | 82.6 | 0.65 | (0.38, 0.87) | | 0.65 | (0.38, 0.87) | |  |  |  |
| We used κ coefficients to evaluate each item rather than the total score. For Understandability and Actionability scores, we calculated Cohen’s κ and the intraclass correction coefficient (ICC). | | | | | | | | | | | | | | | |  |  |  |
