# Supplementary material for: Evaluating fluoride-related YouTube videos in Japan: A comparative analysis of understandability, actionability, and reliability between pro- and anti-fluoride content
Source: PEC Innov. 2026 Feb 8;8:100458. doi: 10.1016/j.pecinn.2026.100458 (PMC12914852; doi:10.1016/j.pecinn.2026.100458)
Supplement: Supplementary file 9 — Supplementary material 9 [file mmc9.docx]

| **Appendix 8. Item-level inter-rater reliability results for items in the GQS** | | | | |  |  |  |
| --- | --- | --- | --- | --- | --- | --- | --- |
|  | % Agree | Cohen’s κ | (95% CI) | Gwet’s AC1 | (95% CI) | ICC | (95% CI) |
| Global Quality Score | 56.5% | 0.45 | (0.18, 0.70) | 0.46 | (0.19, 0.71) | 0.79 | (0.58, 0.91) |
