# Supplementary material for: Evaluating fluoride-related YouTube videos in Japan: A comparative analysis of understandability, actionability, and reliability between pro- and anti-fluoride content
Source: PEC Innov. 2026 Feb 8;8:100458. doi: 10.1016/j.pecinn.2026.100458 (PMC12914852; doi:10.1016/j.pecinn.2026.100458)
Supplement: Supplementary file 10 — Supplementary material 10 [file mmc10.docx]

| **Appendix 9. Item-level inter-rater reliability results for items in the mDISCERN** | | |  |  |  |  |  |  |  |  |  |  |
| --- | --- | --- | --- | --- | --- | --- | --- | --- | --- | --- | --- | --- |
|  |  | Score Results (sum of two raters) | | | |  |  |  |  |  |  |  |
| Item # | Item | 1 | (%) | 0 | (%) |  | Cohen’s κ | (95% CI) | Gwet’s AC1 | (95% CI) | ICC | (95% CI) |
| 1 | Is the aim clear, concise, understandable? | 27 | 58.7 | 19 | 41.3 |  | 0.56 | (0.22, 0.90) | 0.58 | (0.24, 0.91) | 0.57 | (0.22, 0.91) |
| 2 | Are sources of information reliable? | 17 | 37.0 | 29 | 63.0 |  | 0.73 | (0.41, 1.00) | 0.76 | (0.49, 1.00) | 0.73 | (0.44, 1.00) |
| 3 | Is the information presented balanced and unbiased? | 18 | 39.1 | 28 | 60.9 |  | 0.46 | (0.05, 0.82) | 0.50 | (0.13, 0.85) | 0.47 | (0.06, 0.82) |
| 4 | Are additional sources of information listed? | 16 | 34.8 | 30 | 65.2 |  | 0.43 | (0.02, 0.78) | 0.52 | (0.14, 0.84) | 0.44 | (0.02, 0.80) |
| 5 | Does the video address areas of uncertainty? | 16 | 34.8 | 30 | 65.2 |  | 0.47 | (0.18, 0.80) | 0.52 | (0.17, 0.86) | 0.48 | (0.15, 0.82) |
